# Calcium Nitrate Supplementation Improves Meat Quality in Hu Sheep via Microbial and Transcriptomic Regulation

**DOI:** 10.3390/ani16020325

**Published:** 2026-01-21

**Authors:** Yuanshu Zheng, Chen Zheng, Kang Sun, Huihui Liu, Huiyu Fan, Yi Wang, Xuan Nan, Lijing An, Faming Pan, Xinji Wang, Guoyan Xu, Ting Liu

**Affiliations:** 1 College of Animal Science and Technology, Gansu Agricultural University, Lanzhou 730070, China; 15352293951@163.com (Y.Z.); mazh_7853@hotmail.com (C.Z.); landusunkang@163.com (K.S.); lhh1181148654@163.com (H.L.); xh18794773498@163.com (H.F.); 2 Lanzhou Agricultural Radio and Television School, Lanzhou 730000, China; wangyits0505@foxmail.com; 3 Lanzhou Animal Disease Prevention and Control Center, Lanzhou 730046, China; nanxuan6150@163.com; 4 Animal Husbandry and Veterinary Station of Nawu Town, Hezuo 747000, China; 15352288425@163.com; 5 Institute of Animal & Pasture Science and Green Agriculture, Gansu Academy of Agricultural Sciences, Lanzhou 730070, China; pan-faming@163.com; 6 Animal Husbandry and Veterinary Workstation of Minqin County, Wuwei 733399, China; mq555df@sina.com; 7 Animal Husbandry and Veterinary Workstation of Datan Town, Minqin County Agriculture and Rural Bureau, Wuwei 733399, China; 13739356100@163.com

**Keywords:** feed additive, ruminant nutrition, nitrogen metabolism, gene expression, gastrointestinal tract

## Abstract

Research has demonstrated that incorporating nitrate into animal feed can effectively decrease methane production in ruminants, though its impact on carcass characteristics and meat attributes in Hu sheep requires further investigation. This experiment examined how a dietary inclusion of 3% calcium nitrate (CN) influenced slaughter parameters, meat properties, gut microbial populations, and host gene regulation in Hu sheep. The study involved sixty healthy male Hu sheep aged 120 days with comparable body weights (31.11 ± 3.39 kg), randomly allocated into two groups: a control group receiving standard feed (CON) and a CN-supplemented group. The trial lasted 60 days, including a 15-day adaptation period and a 45-day formal trial period. They were housed individually and fed twice daily (at 8:00 and 18:00). The findings revealed that CN supplementation notably reduced the water loss rate in the longissimus dorsi muscle (LD), elevated meat color brightness, and enhanced the proportion of polyunsaturated fatty acids (PUFA), particularly n-6 PUFA, along with the n-3/n-6 PUFA ratio. Conversely, it reduced the levels of saturated fatty acids such as myristic acid (C14:0) and oleic acid (C18:1n9t). Additionally, the treatment boosted ruminal Ammoniacal nitrogen content and total short-chain fatty acid production, thereby contributing to energy metabolism in the animals. Microbiological examination demonstrated that CN supplementation led to a decrease in Fibrobacterota and *Methanobrevibacter* populations within the ruminal environment, while promoting the growth of Proteobacteria in the duodenal region. The gene expression profiling of digestive tract tissues showed an increased activity in nitrogen processing genes (including CA4) and oxidative phosphorylation pathways (such as ATP6), indicating an improved metabolic efficiency and acid–base homeostasis in the host animals. These findings demonstrate that CN-enriched diets enhance the carcass characteristics of Hu sheep by modifying intramuscular lipid profiles through gastrointestinal microbial community restructuring and metabolic pathway adjustments. Such modifications affect energy utilization and acid–base equilibrium, ultimately impacting muscle characteristics and adipose tissue distribution, presenting viable approaches for eco-friendly livestock farming practices.

## Simple Summary

Calcium nitrate holds great promise as a feed additive for ruminants. This study aimed to investigate the role and mechanism by which calcium nitrate regulates microbial and genetic pathways to enhance slaughter performance and meat quality in Hu sheep. The results indicate that adding 3% calcium nitrate to the diet improved meat quality and increased slaughter performance through microbial and transcriptomic pathways, providing a promising research direction for enhancing Hu sheep meat quality.

## 1. Introduction

Mutton is popular on the market because of its characteristics of being lean meat and being easy to digest. The economic value of sheep is high; their meat contains high protein. Slaughtering performance and meat quality are influenced by many factors, such as genetics, diet, and feeding management; in particular, the impact of diet on goats is more prominent [1]. Nitrates have the following three notable advantages when used as additives in the diet. First, they compete with H_2_ for CO_2_ in the rumen, reducing it to NH_3_ rather than CH_4_. Second, they supply NH_3_ to promote microbial protein synthesis, thereby enhancing nitrogen utilization efficiency. Finally, they are cost-effective, stable during pelleting, and widely used in agricultural applications. It is particularly important that these changes in nitrogen metabolism and hydrogen flow in the rumen may trigger a series of chain effects beyond methane reduction, including potentially altering the microbial ecosystem and host tissue metabolism and ultimately affecting the physicochemical properties of the meat [2]. When animals consume nitrate-containing feed, both the reduction of nitrates and the synthesis of propionic acid consume hydrogen. Nitrates exhibit a stronger electron competition than carbon dioxide, thereby effectively suppressing intestinal methane production [3]. When methane generation in ruminants is suppressed, the surplus reducing agents (hydrogen) and carbon structures can be utilized for microbial growth and the formation of acetate or propionate. These compounds are subsequently incorporated into the animal’s tissues as protein or fat deposits [4]. Research indicates that decreasing the methane output can enhance the fatty acid profile in animal muscle tissue [5]. Certain investigations report enhanced weight gain rates (4–9% improvement in daily growth metrics) coupled with better feed conversion ratios [2], whereas other trials observe neutral effects or even a decreased meat yield [6]. These variations stem from multiple factors, including fundamental diet composition (grain-based versus fiber-rich feeds), nitrate compound types (calcium versus sodium derivatives), adjustment period length, and the combined influence of sulfur and molybdenum on nitrate metabolic pathways. It is worth noting that most existing studies focus on a single production or emission indicator. As non-protein nitrogen compounds, nitrates undergo microbial reduction in the rumen, initially forming nitrite as an intermediate before complete conversion to ammonia, which subsequently supports microbial protein synthesis [7]. Studies verify that nitrate supplementation strategies can promote the growth of Bacteroidetes microorganisms within intestinal flora populations and induce modifications in the microbial ecosystem composition [8]. These bacterial communities possess the capacity to enhance the immune response and alleviate gut inflammation, which are among the benefits of nitrate supplementation. In addition, the altered rumen environment and nutrient supply can act as signals to regulate host gene expression. For example, changes in the profile of short-chain fatty acids may affect the expression of genes related to nutrient transport and metabolism in the intestinal epithelium and peripheral tissues [9]. Likewise, changes in nitrogen metabolism may influence the expression of genes associated with pH regulation and cellular energy production [10]. Comprehensive mechanistic studies that can simultaneously assess the resulting changes in meat quality attributes, gastrointestinal microbial diversity, and gene expression changes in host metabolic tissues are still lacking, and are crucial for fully explaining how nitrate supplementation affects the final product quality [8]. Based on these observations, we proposed that optimal nitrate levels might influence both carcass characteristics and meat attributes in Hu sheep.

The current research evaluates how adding 3% calcium nitrate to the standard feed of Hu sheep impacts their slaughter outcomes and meat properties. This investigation offers both a scientific rationale and practical significance for using calcium nitrate as a nutritional supplement in ruminant diets.

## 2. Experimental Design

### 2.1. Animal and Experimental Design

The study involved sixty healthy male Hu sheep aged 120 days with comparable body weights (31.11 ± 3.39 kg), which were randomly allocated into two dietary treatment groups. The control group (CON) received a standard basal ration, while the experimental group was provided with the same basal diet supplemented with 3% calcium nitrate (CN). The research spanned 60 days, including an initial 15-day acclimatization period (days 1–15) followed by a 45-day experimental phase (days 16–60). All animals were placed in individual enclosures (each measuring 1.04 m long, 0.94 m wide, and 0.82 m high) and were fed ad libitum, with fresh feed provided twice daily (at 08:00 and 18:00) and water available ad libitum. Daily pen sanitation was maintained throughout the trial period. Dietary components and CN supplementation levels were calculated based on dry matter content (Table 1). From the initial cohort, 30 animals (15 per group) were selected for detailed parameter analysis. The experimental protocol followed ethical standards established by Gansu Provincial People’s Congress regulations. Body weight measurements were obtained using properly calibrated digital weighing equipment.

### 2.2. Sample Collection and Processing

Sample collection strictly followed operational procedures, with samples immediately stored in liquid nitrogen before transfer to the laboratory for cryopreservation at −80 °C. Digestive tract contents (including rumen fluid, duodenal, and colonic contents) were processed for analysis of short-chain volatile fatty acids (SVFAs), ammonia nitrogen levels, and microbial community profiles. Transcriptomic studies were conducted using rumen and duodenal tissue samples. Additionally, longissimus dorsi (LD) muscle samples were collected to evaluate meat quality traits, including amino acid composition and fatty acid analysis.

### 2.3. Determination of Slaughtering Performance and Meat Quality

Feed administration was discontinued 24 h prior to slaughter, and water supply was ceased 2 h beforehand. Immediate weighing prior to slaughter the following day yielded the pre-slaughter live weight, ensuring consistency in live weight measurements. Following slaughter, hair, viscera, hooves, and the head were removed; the resulting weight is termed the carcass weight. The slaughter rate is calculated by dividing the live weight before slaughter by the carcass weight. The head, hooves, heart, liver, spleen, lungs, and kidneys were separated and weighed individually to calculate the organ index. The pH value and color value of the LD were measured at the 45th minute and the 24th hour using a pH meter (PHS-3C, Shanghai Leici Instrument Factory, Shanghai, China) and a portable colorimeter (DC-23D, Spectrum Technology Co., Ltd., Ningbo, China). We simultaneously determined the cooking rate (take a sample of the longest dorsal muscle and weigh it as M_1_; place it in a boiling water bath and heat for 45 min, then let it cool naturally to room temperature and weigh it as M_2_), water loss rate (take 5 g of the LD and label it as M_3_; place 18 layers of filter paper above and below the meat sample; press with a compressor (ZY201800007, Beijing, China) for 5 min, then remove the meat sample and weigh it, recording it as M_4_), drip loss rate (cut the LD into strips measuring 5 × 3 × 2 cm and weigh them, recording the weight as M_5_; hang the meat samples in a sealed plastic bottle and store at 4 °C for 24 h; remove the samples, blot the surface moisture with filter paper, and immediately weigh them, recording the weight as M_6_), shear force (measure shear force values using a C-LM 2 meat tenderizer on cylindrical meat samples with a diameter of 1 cm; the unit is represented in newtons (N)). According to the description by O’Fallon et al. [11], the fatty acid content in LD was measured using a GC 2010 Plus gas chromatograph (Shimadzu, Kyoto, Japan) through gas chromatography, and the fatty acid content in LD 140 was measured using gas chromatography. After pretreatment of the LD muscle samples by acid hydrolysis (6 M HCl, 110 °C, 24 h), the amino acid composition was determined using a fully automated amino acid analyzer (A300, Menneberg, Clausthal-Zellerfeld, Lower Saxony, Germany). It is worth noting that, after thawing, we removed any visible surface fat and connective tissue from the meat. The calculation formula is as follows:

$$\mathrm{slaughter}\;\mathrm{rate}\;\left(\%\right)\;=\frac{\;\mathrm{carcass}\;\mathrm{weight}}{\;\mathrm{live}\;\mathrm{weight}\;\mathrm{before}\;\mathrm{slaughter}}\;\times \;100\%$$

$$organ\;index\left(\%\right)=\frac{\;\mathrm{organ}\;\mathrm{weight}}{\mathrm{live}\;\mathrm{weight}\;\mathrm{before}\;\mathrm{slaughter}}\;\times \;100\%$$

$$\mathrm{cooking}\;\mathrm{rate}\;\left(\%\right)=\frac{M_{2}}{M_{1}}\;\times \;100\%$$

$$\mathrm{water}\;\mathrm{loss}\;\mathrm{rate}\;\left(\%\right)=\frac{M_{3}-M_{4}}{M_{3}}\;\times \;100\%$$

$$\mathrm{drip}\;\mathrm{loss}\;\mathrm{rate}\;\left(\%\right)=\frac{M_{5}-M_{6}}{M_{5}}\;\times \;100\%$$

### 2.4. Determination of Ammonium Nitrogen and SCFAs

Rumen fluid was subjected to centrifugation at 5000 revolutions per minute for a duration of 10 min. Following centrifugation, 1 milliliter of the resulting supernatant was carefully pipetted into a tube containing 4 milliliters of hydrochloric acid solution with a concentration of 0.2 moles per liter. The mixture was then vigorously mixed using a vortex device. We took 0.2 mL of this mixture and sequentially added 1 mL of Solution A (14% sodium salicylate solution containing 0.08 g sodium nitroprusside) and 1 mL of Solution B (0.3 mol/L NaOH solution containing 2 mL sodium hypochlorite). This was mixed thoroughly and incubated for 10 min. We transferred 200 µL of the reaction mixture to a 96-well plate. We measured the absorbance at 700 nm and calculated the ammonia nitrogen concentration using the standard curve. The methods for determining SCFAs refer to our previous studies [12]. Briefly, rumen and colon contents from ruminants were thawed at 4 °C, accurately weighed to 1.0 g, and mixed with 1.0 mL ultrapure water by vortexing. The homogenate was then centrifuged at 5000 rpm for 10 min. From the clarified supernatant, 1 milliliter was carefully withdrawn. This was followed by the addition of 0.2 mL of a 25% metaphosphoric acid solution containing the internal standard solution containing 2-ethylbutyric acid. Following thorough mixing, the preparation was maintained at 0 °C for half an hour before undergoing centrifugation at 10,000× *g* for a duration of 10 min. After centrifugation, the clear liquid phase was carefully separated and subjected to analytical examination using an Agilent 6890N gas chromatography (Agilent Technologies, Santa Clara, CA, USA) system fitted with an HP19091N-213 separation column.

### 2.5. Microbial Diversity Analysis

Rumen fluid and duodenal and colonic contents were collected from Hu sheep and transported under cryogenic conditions to Novogene Co., Ltd. (Beijing, China) for microbial community analysis through 16S rDNA sequencing. The experimental procedure involved extracting total genomic DNA through an adjusted CTAB method, followed by quality assessment using both gel electrophoresis and Nanodrop™ 2000 spectrophotometric analyzer (Thermo Fisher Scientific, Wilmington, MA, USA). After diluting the DNA to 1 μg/mL, we amplified the V3–V4 region of the bacterial 16S rRNA gene using primers (515F: (5′-GTGCCAGCMGCCGCGGTAA-3′) and 806 R: (5′-GGACTACHVGGGTWTCTTAAT-3′)) [13]. Amplification products underwent verification via 2% agarose gel electrophoresis, followed by purification with Qiagen extraction kits and quantification using Qubit^®^ 2.0 technology. Library preparation was conducted with the TruSeq^®^ DNA PCR-Free Kit (Illumina, San Diego, CA, USA), incorporating uniquely indexed adapters. Sequencing quality was ensured through the Illumina NovaSeq 6000 platform (Illumina, San Diego, CA, USA) using 2 × 250 bp paired-end sequencing. Read merging was accomplished with FLASH v1.2.7 software [14]. After quality filtering, demultiplexing was performed using QIIME v1.9.1 [15,16]. Chimeras were removed by comparing sequences against the SILVA Gold database using VSEARCH (version 2.22.1). OTUs were clustered at 97% similarity using the UPARSE algorithm (USEARCH version 11.0) [17,18]. Species annotation was performed using the SILVA SSURef NR138 data base [19]. Sequence alignment and phylogenetic analysis were performed using MUSCLE v3.8.31 [20].

### 2.6. Transcriptome Analysis

Randomly selected rumen and duodenal samples from each treatment group underwent transcriptomic analysis. RNA isolation was performed utilizing TRIzol reagent (Life Technologies, Carlsbad, CA, USA), followed by quantification and quality assessment using the NanoDrop-2000 spectrophotometer (NanoDrop Technologies, Wilmington, DE, USA). RNA concentration and integrity were assessed using a Qubit 2.0 Fluorometer (Life Technologies) and a Bioanalyzer 2100 system (Agilent Technologies, Santa Clara, CA, USA). Only samples with an RNA Integrity Number (RIN) ≥ 7.0 were used for library preparation. RNA-Seq libraries were constructed using the TruSeq Stranded mRNA Kit (Illumina, San Diego, CA, USA) and subjected to 150 bp paired-end sequencing on the Illumina HiSeq 4000 platform. Sequencing was entrusted to Beijing Novozymes Technology Co. (Beijing, China). To mitigate the risk of false positives due to the small sample size (n = 3), a rigorous analysis workflow was employed. Differential expression analysis was performed using DESeq2 (v1.20.0), with low-count genes filtered out through independent screening. Following Benjamini–Hochberg correction, a dual threshold of FDR < 0.05 and |log_2_FoldChange| ≥ 1 was applied to identify significantly differentially expressed genes, thereby maintaining strict control over potential false-positive findings [21,22].

### 2.7. Statistical Analysis

Experimental data were systematically compiled using Microsoft Excel and subjected to statistical evaluation through independent two-sample *t*-tests conducted in IBM SPSS Statistics version 26.0.

Gene expression differences across comparative groups were examined utilizing the DESeq2 package (version 1.20.0); genes with *p* < 0.05 were classified as differentially expressed genes. Functional annotation of these genes was carried out through Gene Ontology (GO) classification and KEGG pathway mapping using the clusterProfiler tool (version 3.4.4). Differential expression analysis between the two comparison combinations was performed using DESeq2 software (1.20.0). Genes with *p* < 0.05 were classified as differentially expressed genes. GO enrichment analysis and KEGG pathway enrichment analysis of differentially expressed genes were performed using clusterProfiler (3.4.4) software.

Associations between gut microbiota composition and SCFA profiles were investigated employing Spearman’s rank correlation method, with statistical significance determined at the conventional *p* < 0.05 level.

## 3. Results

### 3.1. Slaughtering Performance and Meat Quality

**Figure 1:**
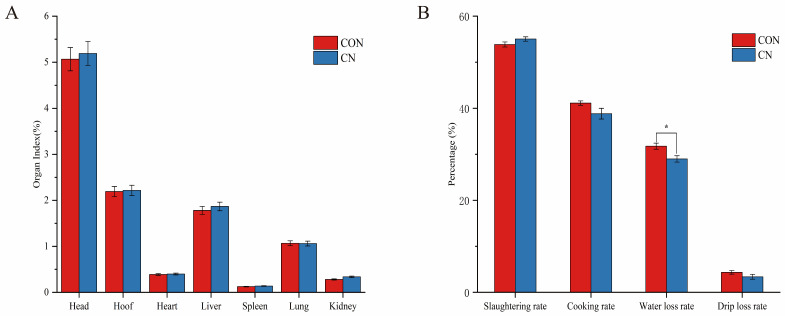
Effect of adding 3% calcium nitrate to the diet on the organ index (**A**) and slaughtering rate and meat quality (**B**) of Hu sheep. * *p* < 0.05.

Figure 1 indicates that the CN group had an increased slaughter rate and decreased cooking rate, water loss rate, and drip loss rate; in particular, there was a significant difference between the water loss rate of the control and experimental groups (*p* < 0.05) (Figure 1B). There were no substantial variations between the CON group and the CN group for the other labels. There were no substantial variations in the organ indices of the head, hooves, heart, liver, spleen, lungs and kidneys between CON and CN groups, but it can be seen that the addition of CN to the basal diet basically improved the organ indices compared to the CON group (Figure 1A).

At the 45th minute, the lightness of the CN group’s meat was significantly higher than that of the control group (*p* < 0.001), while there were no differences in redness and yellowness between the groups. At 24 h, the meat color values of all groups increased, with no significant differences between the groups. At both time points, the pH values of the CN group were significantly lower than those of the control group (*p* < 0.001), and its shear force was also significantly higher (*p* < 0.001). (Table 2).

The data presented in Table 3 demonstrate that a dietary supplementation with calcium nitrate led to notable alterations in the fatty acid composition of Hu sheep’s longissimus dorsi muscle. Specifically, this intervention caused a marked decrease in the levels of myristic acid (C14:0), Pentadecanoic acid (C15:0), Heptadecanoic acid (C17:0), and Elaidic acid (C18:1n9t) (*p* < 0.05). Conversely, significant increases were observed in the concentrations of Capric acid (C10:0), Pentadecenoic acid (C15:1), Linolelaidic acid (C18:2n6t), Linoleic acid (C18:2n6c), and Heneicosanoic acid (C21:0), along with polyunsaturated fatty acids (PUFA), n-6 PUFA, and the n-3/n-6 PUFA ratio (*p* < 0.05). However, the treatment showed no statistically significant impact on the remaining intramuscular fatty acids.

### 3.2. Ammoniacal Nitrogen and SCFAs

**Figure 2:**
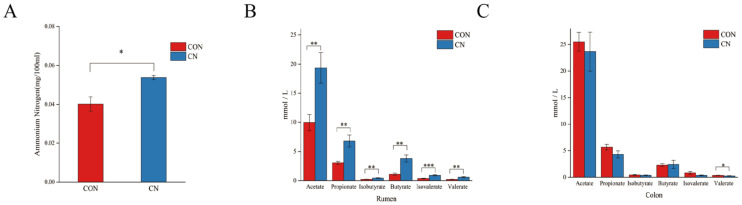
Effect of adding 3% calcium nitrate to the diet of Hu sheep on the fermentation parameters of the rumen and colon. (**A**) The effect of calcium nitrate on the rumen ammonia nitrogen of Hu sheep. (**B**) Effects of calcium nitrate on short-chain volatile compounds in rumen contents. (**C**) Effects of calcium nitrate on short-chain volatile compounds in colon contents. * *p* < 0.05, ** *p* < 0.01, *** *p* < 0.001.

The data presented in Figure 2 demonstrate notable differences between the CN and CON groups. The CN group had a significantly increased ammonia nitrogen content in the rumen (*p* < 0.05, Figure 2A). The addition of CN to the basic diet significantly increased the levels of acetic acid, propionic acid, isobutyric acid, butyric acid, valeric acid, and isovaleric acid in the rumen (*p* < 0.01), with a particularly significant difference in valeric acid content (*p* < 0.001, Figure 2B). Conversely, in the colon, the CN group displayed significantly lower valeric acid levels relative to the CON group (*p* < 0.05, Figure 2C).

### 3.3. Digestive Tract Microbial Community

#### 3.3.1. Alpha Diversity Analysis

**Figure 3:**
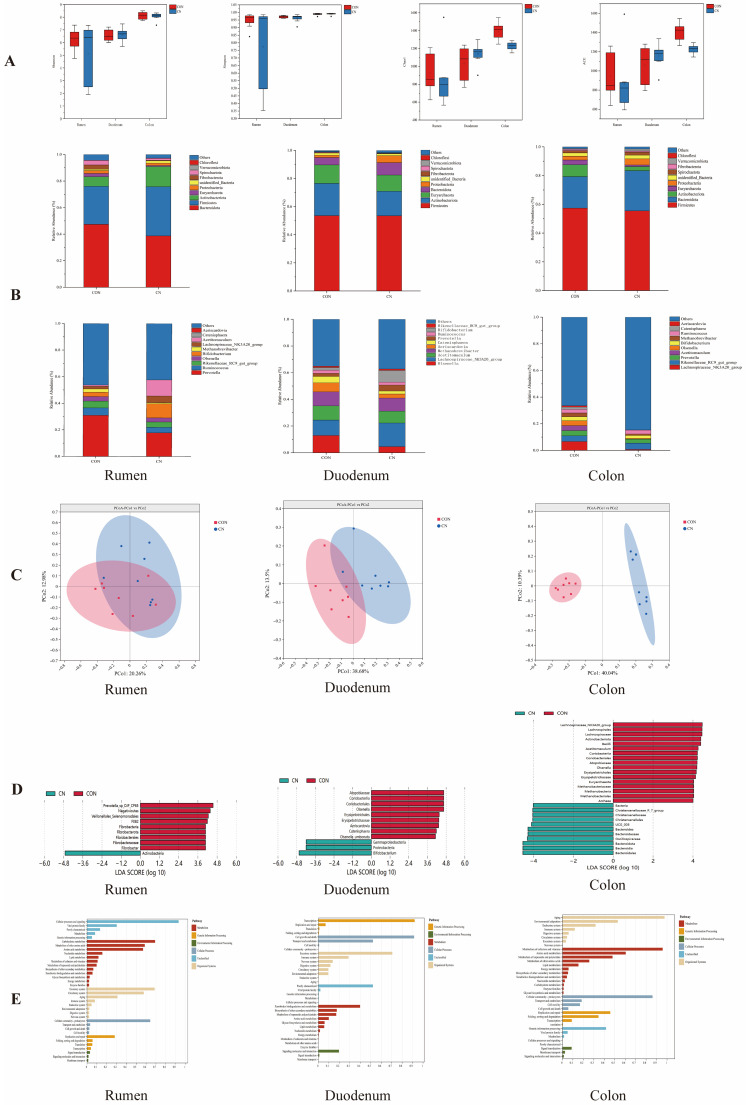
Effect of adding 3% calcium nitrate to the diet on the microbial diversity and functional prediction of the gastrointestinal tract of Hu sheep. (**A**) Shannon, Simpson, ACE, Chao1. Alpha Diversity analysis index for different samples at 97% consistency thresholds. (**B**) The relative abundance of the top 10 gastrointestinal microbes at the phylum level and genus level. (**C**) Gastrointestinal PCoA. Beta Diversity is a comparative analysis of microbial community composition across different samples. If the samples are closer together, it means that the species composition is more similarly structured, so samples with a high similarity in community structure tend to cluster together, and samples with very different communities are far apart. (**D**) Analysis of LEfSe differences in gastrointestinal tract. LEfSe revealed distinct microbial signatures between the treatment groups. The magnitude of the LDA horizontal axis value indicates the extent to which microbial taxa contribute to differences between groups (the larger the absolute value, the more significant the contribution; LDA value was greater than or equal to 2). (**E**) Prediction of gastrointestinal microbiota functions. Based on database annotation results, functional prediction of microbial communities in samples was performed using Tax4Fun software (version 1.0, Berlin, Germany).

As can be seen from Figure 3A, there was no significant difference between the CN and CON groups.

#### 3.3.2. Relative Abundance of Species

The effect of the addition of CN to the basal diet on the microbial composition of the gastrointestinal tract, as determined by an analysis of microorganisms at the phylum and genus levels, is shown in Figure 3B.

At the phylum classification, the rumen microbiota was primarily composed of Bacteroidota and Firmicutes, and the CN group increased the relative abundance of Actinobacteria and Firmicutes and decreased the relative abundance of Fibrobacterota in the rumen compared to the CON group. In the duodenum, Firmicutes, Actionbacteriota, and Euryarchaeotad were the dominant flora, and the CN group had a decreased relative abundance of Actinobacteriota and Euryarchaetoa and increased relative abundance of Bacteroidota and Proteobacteria in the duodenum compared to the CON group. The colonic microbiota was dominated by Firmicutes and Bacteroidota, and the CN group had a decreased relative abundance of Actinobacteriota and Euryarchaetoa and increased relative abundance of Bacteroidota and Proteobacteria in the colon.

At the genus level, in the rumen, *Prevotella* was the dominant flora, and the CN group had an increased relative abundance of *Acetitomaculum* and Bifidobacterium and decreased relative abundance of *Olsenella*, *Ruminococcus*, and *Methanobrevibacter* in the rumen compared to the CON group. Within the duodenal microbiota, *Olsenella* and *Lachnospiracea* constituted the principal bacterial communities. The CN dietary regimen demonstrated significant modulatory effects, diminishing the relative quantities of *Olsenella*, *Actitomaculum*, *Aeriscardovia*, *and Methanobrevibacter*, while enhancing *Prevotella* populations in duodenal samples relative to the control group. In the colon, *Lachnospiraceae* was the dominant flora, and the CN group had a decreased relative abundance of *Lachnospiraceae* and *Methanobrevibacter* and in the colon.

#### 3.3.3. Beta Diversity Analysis

The microbial populations in the rumen showed minimal variation between the CON and CN groups, with the CN intervention failing to produce notable alterations in the rumen microbiota composition. The addition in the CN group induced a restructuring of the duodenal microbial community, resulting in significant differences compared to the CON group. The PCoA demonstrated a significant difference in the microbial community structure between the two groups; thus, the CN group significantly altered the colonic microbial community (Figure 3C).

#### 3.3.4. Microbial Communities of the Gastrointestinal Tract

In the rumen, the CON group was significantly increased in *Fibrobacter* and the CN group was significantly increased in Actinobacteria. In the duodenum, the CON group was significantly increased in *Olsenella*, *Aeriscardovia*, and *Catenisphaera*, and the CN group was significantly enriched in Bifidobacterium. In the colon, the CON group was significantly increased mainly in *Olsenella* and *Acetitomaculum*, and the CN group was significantly increased mainly in Bacteroidaceae (Figure 3D).

#### 3.3.5. Prediction of Gastrointestinal Microbial Function in Hu Sheep

We predicted the functions of the gut microbiota in Hu sheep across the rumen, duodenum, and colon. The results indicate that the predicted functions were primarily enriched in categories such as metabolism, genetic information processing, environmental information processing, cellular processes, and the organismal system. These results indicated that the microbiota functions were similar in the six groups, but there were some differences in the relative abundance, and the main enrichment function was metabolism (Figure 3E).

### 3.4. Transcriptome Analysis

#### 3.4.1. Analysis of Differentially Expressed Genes in the Transcriptome

**Figure 4:**
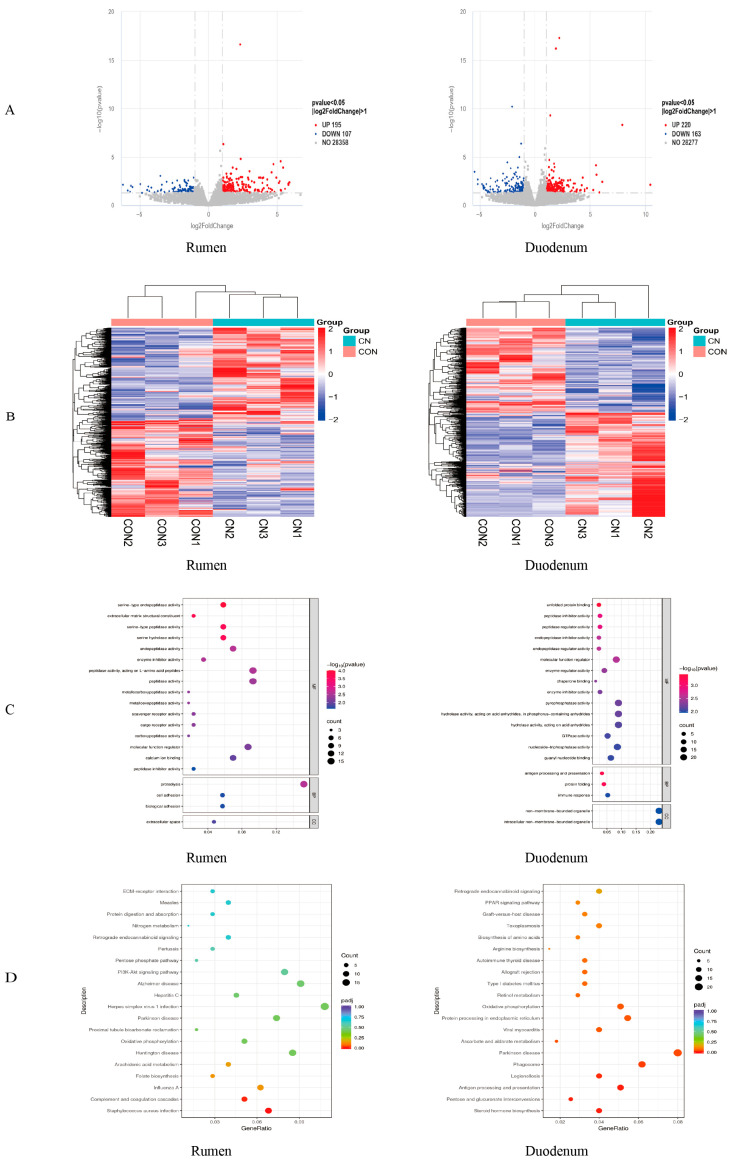
(**A**) Analysis of differentially expressed genes in the rumen and duodenum of Hu sheep. |log2FoldChange| > 1 and *p* value < 0.05 are used as the standard. (**B**) Heatmap of differentially expressed genes clustering in the rumen and duodenum. In heatmaps, samples with similar expression patterns are clustered together. The color in each cell reflects the normalized expression value obtained after row-wise normalization of the expression data. (**C**) GO analysis of differentially expressed genes in the rumen and duodenum of Hu sheep. To clarify the functions of differentially expressed genes in different diets, the differentially expressed genes in the rumen of Hu sheep in the control group and the experimental group were annotated by Biological Process (GO-BP), Cellular Component (GO-CC), and Molecular Function (GO-MF). (**D**) Annotation of KEGG function of gastrointestinal microbes in Hu sheep. From the KEGG enrichment results, the top 20 most significant KEGG pathways were selected and visualized using scatter plots.

As can be seen from Figure 4, a total of 302 differential genes were screened in the rumen of Hu sheep in the CON group and the CN group, among which 195 genes were up-regulated and 107 genes were down-regulated. A total of 383 differential genes were screened in the duodenum, among which 220 genes were up-regulated and 163 genes were down-regulated (Figure 4A). Figure 4B shows that the biological replicates of the same group of three samples are clustered in the same family, indicating that the samples of the same group collected in the experiment have a high accuracy and reliability.

#### 3.4.2. GO Feature Enrichment Analysis

Gene Ontology (GO) enrichment analysis was performed on the rumen and duodenum of Hu sheep. In the rumen tissue, the biological process (BP) category revealed that these genes were predominantly associated with proteolysis, cell adhesion, and bioadhesion. In the cellular component (CC), differentially expressed genes play a major role in the extracellular space. The molecular function (MF) mainly involves serine-type endopeptidase activity, extracellular matrix structural constituent, serine-type peptidase activity, serine hydrolase activity, endopeptidase activity, enzyme inhibitor activity, peptidase activity acting on L-amino acid peptides, peptidase activity, metallocarboxypeptidase activity, metalloexopeptidase activity, scavenger receptor activity, cargo receptor activity, carboxypeptidase activity, molecular function regulation, calcium ion binding, and peptidase inhibitor activity. Differentially expressed genes in the duodenum were subjected to Gene Ontology enrichment analysis. It was found that the differentially expressed genes in BP mainly play a role in antigen processing and presentation, protein folding, and immune response. In CC, differentially expressed genes play a major role in non-membrane-bounded organelles and intracellular non-membrane-bounded organelles. MF mainly involves unfolded protein binding, peptidase inhibitor activity, peptidase regulator activity, endopeptidase inhibitor activity, endopeptidase regulator activity, molecular function regulation, enzyme regulator activity, chaperone binding, enzyme inhibitor activity, pyrophosphatase activity, hydrolase activity acting on acid anhydrides and in phosphorus-containing anhydrides, hydrolase activity acting on acid anhydrides, GTPase activity, nucleoside–triphosphatase activity, and guanyl nucleotide binding (Figure 4C).

#### 3.4.3. KEGG Pathway Enrichment Analysis

An analysis of KEGG pathways for differentially expressed genes in Hu sheep was conducted. The investigation revealed the twenty most prominently enriched pathways within the rumen, which encompassed ECM-receptor interactions, Measles Protein digestion and absorption, nitrogen metabolism, Retrograde endocannabinoid signaling, Pertussis, the pentose phosphate pathway, the PI3K-Akt signaling pathway, Alzheimer disease, Hepatitis C, Herpes simplex virus 1 infection, Parkinson’s disease, the PI3K-Akt signaling pathway, Proximal tubule bicarbonate reclamation, oxidative phosphorylation, Huntington disease, Arachidonic acid metabolism, Folate biosynthesis, Influenza A, complement and coagulation cascades, and Staphylococcus aureus infection. Among them, the main signaling pathways related to metabolism include nitrogen metabolism and oxidative phosphorylation (Figure 4D). There were two differential genes for nitrogen metabolism: *CA4* was up-regulated and *CA2* was down-regulated. There were nine differential genes for oxidative phosphorylation, among which the up-regulated genes were *ND5*, *ND3*, *ND2*, *SDHD*, *NDUFA11*, and *LOC101121518*, and the down-regulated genes were *ND4L*, *COX2*, and *ND6*.

The duodenal mucosa analysis identified 20 notably enriched biological pathways, with prominent metabolic associations observed in pentose and glucuronate interconversions and oxidative phosphorylation (Figure 4D). There were 14 differentially expressed genes in the oxidative phosphorylation pathway: the expressions of *ND1*, *NDUFA4*, *ND3*, *LOC114114909*, *NDUFA6*, *COX1*, *LOC114110816*, *COX1*, *ND4L*, *ATP6*, *ND5*, and *ND6* were up-regulated, and *ND4* and *ND4L* expressions were down-regulated. There were seven differentially expressed genes in the pentose and glucuronate interconversions: *LOC101117163*, *SORD*, and *UGT1A3* expression was up-regulated; *KL*, *UGT1A3*, *AKR1A1*, and *LOC101122788* expression was down-regulated.

### 3.5. Correlation Analysis of Differential SCFAs and Microbial Abundance

**Figure 5:**
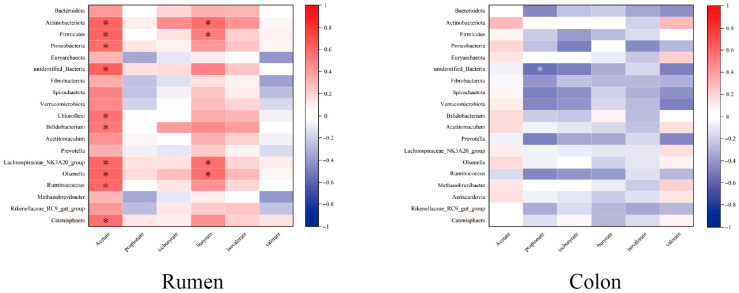
Correlation analysis between rumen and duodenum microorganisms and SCFAs. The depth of color is proportional to the correlation values. * *p* < 0.05.

To investigate the relationship between SCFAs and bacterial communities in the rumen and colon, we performed Spearman’s correlation analysis (Figure 5). In the rumen, the concentration of acetate is positively correlated with Actinobacteriota, Firmicutes, Proteobacteria, Chloroflexi, *Bifidobacterium*, *Olsenella*, *Ruminococcus*, and *Catenisphaera* (*p* < 0.05), while the concentration of butyrate is positively correlated with Actinobacteriota, Firmicutes, and *Olsenella* (*p* < 0.05). In the colon, there is basically no difference in short-chain volatile fatty acids and microorganisms.

## 4. Discussion

### 4.1. Meat Quality and Muscle Fat Deposition

In our study, supplementing the diet with CN did not significantly affect the slaughtering performance of Hu sheep, which is consistent with the findings of Araujo and indicates that calcium nitrate may serve as a promising nitrogen source [23]. Additionally, the results revealed significant differences in both the water loss rate and shear force, with the CN group showing a lower water loss rate. A decrease in water loss is typically associated with an improved meat quality. The study further found that muscles with reduced water loss exhibited a superior eating quality [24]. Significant differences in meat pH were observed both 45 min and 24 h post-slaughter, with a decrease in pH values in the CN group. Nitrates may indirectly influence muscle metabolism (e.g., lactic acid production) by affecting rumen fermentation and energy metabolism [25]. The deposition of intramuscular fat in animals is a complex process influenced by various factors, including breed, sex, age, dietary nutrient levels, and deposition site [26,27,28]. As dietary guidelines increasingly emphasize a higher intake of n-3 polyunsaturated fatty acids, lamb has become an important contributor of these beneficial lipids in human nutrition. Both linoleic acid and α-linolenic acid are indispensable fatty acids for humans, serving as the metabolic precursors for the n-6 and n-3 polyunsaturated fatty acid families, respectively [29]. The results of the study demonstrated that the dietary inclusion of calcium nitrate significantly enhanced the linoleic acid content, thereby potentially facilitating the endogenous synthesis of polyunsaturated fatty acids. Therefore, the addition of calcium nitrate to the ration significantly elevated PUFA, n-6 PUFA, and n-3/n-6 PUFA in the longest dorsal muscle of the Hu sheep. Pascual et al. concluded that PUFA had an ab initio synthesis inhibitory effect on SFA and MUF [30]. Reducing the ratio of SFA (e.g., C16:0, C18:0) in the muscle of ruminant animals reduces the intake of dietary LDL-C in humans, reducing the risk of cardiovascular disease. SFA promotes hepatic lipogenesis through the activation of SREBP-1 [31], whereas PUFA (e.g., n-3) inhibits this pathway and improves fatty liver [32]. In this study, a dietary supplementation with calcium nitrate significantly reduced the concentrations of C14:0, C15:0, C17:0, C18:1n9t, and total saturated fatty acids in the LD of Hu sheep, consistent with the findings reported by Pascual et al. [30]. Furthermore, C16:0, C18:0, and C18:1n9c were the predominant fatty acids in the longissimus dorsi of Hu sheep, collectively comprising approximately 78% to 87% of the total fatty acid content [33,34]. The present results align with these findings.

### 4.2. Ammonia Nitrogen and SCFAs in the Gastrointestinal Tract

Rumen ammonia nitrogen is produced through the microbial degradation of dietary crude protein, peptides, amino acids, and non-protein nitrogen (e.g., urea, nitrates) within the rumen. It serves as the primary nitrogen source for microbial protein synthesis and provides nitrogenous skeletons for volatile fatty acid formation, thereby influencing host energy supply and performance [34]. The CN group increased the concentration of ammonia nitrogen in the rumen fluid by 27%. This result aligns with the classic reduction pathway of nitrates in the rumen, NO_3_^−^ → NO_2_^−^ → NH_3_, where each mole of nitrate consumes 4 moles of metabolic hydrogen and releases 1 mole of NH_3_ [35]. Since this process preferentially competes for H_2_, it inhibits the reduction of CO_2_ to CH_4_. The conserved reducing equivalents not only enhance the total volatile fatty acids but also promote more ammonia nitrogen entering the microbial protein synthesis pathway, thereby improving nitrogen utilization efficiency [36]. Microorganisms in the rumen (primarily bacteria) anaerobically ferment the structural carbohydrates in feed (such as cellulose and hemicellulose), producing short-chain volatile fatty acids, acetic acid, propionic acid, and butyric acid as the primary components, collectively accounting for over 90% of the total [37]. Supplementing the diet with CN significantly elevated the concentration of SCFAs, mainly due to the enrichment of typical cellulose-degrading bacteria and key nitrate-reducing microbes, which enhanced the fermentation of structural carbohydrates. Research indicates that the addition of 1–2% nitrate to bull diets leads to a linear increase in total VFA and acetic acid concentration, consistent with the results of this study [38].

### 4.3. Microbial Diversity of the Gastrointestinal Tract and Gene Expression

Rumen microorganisms play a key role in methane production, and about 78% are hydrogenotrophic methanogenic bacteria, which use hydrogen and carbon dioxide to produce methane [39].

The current study revealed that Bacteroidota and Firmicutes constituted the predominant bacterial phyla in both the rumen and colon, while the duodenum exhibited a distinct microbial composition dominated by Actionbacteriota, Firmicutes, and Euryarchaeotad. These findings align with previous research by Perumbakkam et al., who identified Bacteroidota and Firmicutes as the most prevalent bacterial groups in ruminant rumen ecosystems [40]. The addition of calcium nitrate to the ration significantly reduced the abundance of Fibrobacterota in the rumen and Actionbacteriota and Euryarchaeotad in the colon, and significantly elevated the abundance of Proteobacteria in the duodenum. Nitrate is reduced to nitrite (NO_2_^−^) and nitric oxide (NO) in the rumen, and Marais, J P et al. showed that the major cellulolytic bacteria commonly found in the rumen were severely inhibited by nitrite, similar to the results of the present trial [41].

At the genus level, the trial results indicated that a dietary supplementation with CN significantly reduced the ruminal abundance of *Methanobrevibacter* and *Methanosphaera*. Asanuma, N., showed that the number of methanogenic bacteria was greatly reduced due to the presence of nitrate in the diet [42]. Popova, M., showed that nitrates reduced rumen methanogenic bacteria activity. The CN group had a reduced abundance of *Olsenella* in the rumen, a result that is similar to the findings of Bharanidharan R et al. [43]. Significantly, there was a decline in the abundance of *Methanosphaera* in the duodenum. Patra and Yu et al. concluded that methanogens draw energy for their growth and development through the process of methanogenesis, and if methane inhibitors are used the production of methane will be inhibited, thus cutting off the energy source of methanogens and inhibiting the growth of methanogen [44]. Zhou et al. concluded that nitrate can compete with methanogens for hydrogen, which leads to the blockage of methane synthesis [45]. Previously, it was suggested that nitrate supplementation increased nitrate-reducing bacteria and decreased the metabolic activity of rumen methanogenic bacteria [8].

The differentially expressed gene KEGG pathway was analyzed in the control group and the experimental group of the rumen tissue of Hu sheep, and it was found that the main signaling pathways related to metabolism were nitrogen metabolism and pentose phosphate. *CA4* is an up-regulated gene and *CA2* is a down-regulated gene in the nitrogen metabolism pathway. *CA4* and *CA2* catalyze the reversible hydration of carbon dioxide into bicarbonate and protons [10], and are therefore essential for maintaining intracellular and extracellular pH, and nitrate (NO_3_^−^) is reduced to nitrite (NO_2_^−^) and ammonia (NH_3_) by microorganisms in the rumen, which may affect rumen pH and in turn regulate gene expression. When calcium nitrate is added to the diet, rumen microorganisms (e.g., denitrifying bacteria) gradually reduce NO_3_^−^ to nitrite (NO_2_^−^), nitric oxide (NO), nitrous oxide (N_2_O), and nitrogen (N_2_), and the consumption of H^+^ can theoretically increase rumen pH [46]. The host may up-regulate *CA4* to increase H^+^ production and maintain acid–base balance, similar to the experimental results. Through this process, the rumen pH balance is maintained. In addition, *DERA* was up-regulated in the pentose phosphate pathway, and *TKT* and *PFKM* were down-regulated. *DERA* (2-Deoxyribose-5-Phosphate Aldolase, 2-deoxyribose-5-phosphoaldolase) exerts core catalytic functions in both the pentose phosphate cycle and purine/pyrimidine metabolism [47].

Glyceraldehyde-3-phosphate (G3P) links the pentose phosphate pathway to glycolysis and enters energy metabolism. G3P is glycolyzed to produce pyruvate, which eventually enters the tricarboxylic acid cycle (TCA) or gluconeogenesis [48]. *PFKM* is an ATP-dependent 6-phosphofructokinase; muscle-type *PFKM* catalyzes ATP’s phosphorylation of D-fructose-6-phosphate to fructose-1,6-bisphosphate, which is the first step in glycolysis and regulates carbon flow to energy metabolism (ATP production) or storage (e.g., fat synthesis). It provides energy to the lamb and promotes the growth of its muscle and fat. *PFKM* deficiency causes a rare metabolic muscle disorder called Tarui disease, also known as glycogen storage disease type VII (GSD VII) [49,50].

The KEGG pathway of differentially expressed genes was analyzed in the CON and CN groups in the duodenum tissues of Hu sheep, and the main signaling pathways related to metabolism were found to be the interconversion pathway of pentose and glucuronide and oxidative phosphorylation. The main up-regulated genes in the pentose and glucuronate interconversions of pentose and glucuronide were *LOC101117163*, *SORD*, and *UGT1A3*, and the down-regulated genes were *AKR1A1* and *KL*. *AKR1A1* is an aldose reductase dependent on NADPH, and *AKR1A1* is involved in the oxidative reaction that catalyzes the generation of aldose from sugar alcohols to produce NADPH, which causes glutathione (GSH) regeneration to counteract oxidative stress [51]. GSH and others retard myoglobin oxidation and maintain redness. Consistent with the experimental results, the addition of calcium nitrate slightly increased the redness of the meat color, which could improve the meat quality. When calcium nitrate is added to the diet, NO_3_-reduction depletes NADPH, and *AKR1A1* activity may be down-regulated, similar to the results of this experiment, so it is worth noting that additional antioxidant supplementation is required when calcium nitrate is added to the diet. The *SORD*-encoded enzyme catalyzes the conversion of sorbitol to fructose [52]. Fructose can be further metabolized to energy (ATP), which is supplied in the liver, muscle, and adipose tissues of sheep to promote muscle and fat growth. *KL* maintains calcium and phosphorus through a synergistic multiorgan action, and its functional deficiencies are closely associated with metabolic bone disease, vascular calcification, and aging [53]. Calcium overload when feeding calcium nitrate may interfere with phosphorus homeostasis. Up-regulated genes in the oxidative phosphorylation pathway include *ATP6*, *COX1*, etc. *ATP6* encodes the Fo subunit of ATP synthase, which is directly involved in ATP synthesis and uses the proton gradient (H^+^ transmembrane potential difference) to drive the combination of ADP and inorganic phosphoric acid (Pi) to generate ATP, which provides energy for the cell [54]. The *ATP6* gene has a direct impact on the growth, reproduction, and metabolic health of the sheep by regulating mitochondrial energy synthesis, resistance, and metabolic health. *COX1* is involved in the oxidative phosphorylation (OXPHOS) pathway, generating ATP to energize the body. The expression of all these genes is affected by factors such as diet and age, and increases gradually with age, remaining stable as the lamb matures [55].

### 4.4. Correlation Analysis of Differential SCFAs and Microbial Abundance

In the rumen, the concentration of acetate is positively correlated with Actinobacteriota, Firmicutes, Proteobacteria, Chloroflexi, *Bifidobacterium*, *Olsenella*, *Ruminococcus*, and *Catenisphaera*. Research indicates that adding 2.5% calcium ammonium nitrate significantly increases the proportion of acetate in the diet, and acetate is positively correlated with *Ruminococcus*, consistent with the results of this experiment [43]. The aforementioned microbial communities utilize key enzymes such as Cel48A cellulase, the F6PPK bifid pathway, the 3-HP CO_2_ fixation pathway, and nitrate reductase to convert cellulose, lactic acid, or nitrates into acetate, butyrate, or NH_3_. This illustrates that these microorganisms are positively correlated with acetate, which is absorbed in the rumen and enters the liver and peripheral tissues via the portal vein. In the mitochondria, it is oxidized to acetyl-CoA and enters the tricarboxylic acid cycle, generating a net of 10 mol ATP per 1 mol, meeting 60–70% of the animal’s maintenance energy requirements [56].

## 5. Conclusions

This study reveals the synergistic mechanism through which the dietary supplementation of 3% calcium nitrate improves meat quality in Hu sheep, mediated by a modulation of the ‘rumen microbiota–host gene’ interaction network. The supplementation optimized rumen fermentation, increasing the supply of ammonia nitrogen and short-chain fatty acids, while suppressing methanogenic populations and promoting beneficial bacteria. At the molecular level, calcium nitrate up-regulated key genes in nitrogen metabolism (CA4) and oxidative phosphorylation (ATP6) pathways, thereby enhancing energy metabolism and the acid–base balance. These changes collectively improved the muscle water-holding capacity and increased the proportion of polyunsaturated fatty acids (particularly n-6 PUFAs), thus enhancing both the eating quality and nutritional value of the meat. This work provides important evidence for the application of calcium nitrate in achieving sustainable ruminant production with high-quality outputs.

## Figures and Tables

**Table 1:** Dietary composition and chemical composition (dry matter basis).

Item	Treatment ^a^	
	CON	CN
Ingredient composition, % of DM		
Corn stalks	17.76	17.76
Soybean hulls	10.34	10.34
Sunflower skin	2.00	2.00
Corn	48.60	49.65
Soybean meal	11.35	7.55
Sesame cake	3.00	3.00
Molasses	5.00	5.00
Stone powder	0.25	0
NaCl Sodium chloride	0.70	0.70
Premix ^b^	1.00	1.00
Calcium nitrate	0.00	3.00
Total	100	100
Chemical composition ^c^		
Dry matter, % (as-fed basis)	90.25	90.50
Digestible energy, MJ/kg of DM	12.31	11.92
Crude protein, % of DM	14.34	14.70
Ether extract, % of DM	2.48	2.95
Neutral detergent fiber, % of DM	39.22	38.09
Acid detergent fiber, % of DM	17.20	16.89
Calcium, % of DM	0.31	0.81
Phosphorus, % of DM	0.21	0.22

^a^ Treatment; CON means control group, CN means 3% calcium nitrate group. ^b^ Premix ingredients: iron Fe, 25 mg; manganese Mn, 40 mg; zinc Zn, 40 mg; copper Cu, 8 mg; iodine I, 0.3 mg; selenium Se, 0.2 mg; cobalt Co, 0.1 mg; vitamin A VA, 940 IU; vitamin D VD, 111 IU; vitamin E VE, 20 IU. ^c^ Chemical composition was calculated based on the analyzed values of individual feed ingredients.

**Table 2:** Effect of adding calcium nitrate to the diets of Hu sheep on the shear force meat quality.

Item	Treatment ^a^		SEM ^b^	*p*-Value
	CON	CN		
Color_45min_ ^c^				
L*	18.47	19.82	0.205	<0.001
a*	2.95	3.29	0.094	0.072
b*	36.51	37.21	0.304	0.200
Color_24h_				
L*	22.00	20.32	0.312	0.005
a*	8.67	7.69	0.350	0.162
b*	38.17	37.46	0.305	0.250
Meat pH				
pH_45min_	7.23	6.74	0.06	<0.001
pH_24h_	6.19	5.67	0.06	<0.001
Shear force (N)	65.60 ^b^	87.10 ^a^	3.34	<0.001

^a^ Treatment; CON means control group, CN means 3% calcium nitrate group. ^b^ SEM means standard error. ^c^ Color_45min_: L*, brightness of color; a*, redness; b*, yellowness.

**Table 3:** Effect of adding 3% calcium nitrate to the diets of Hu sheep on the fatty acids of the longest dorsal muscle (%).

Item	Treatment ^a^		SEM ^b^	*p*-Value
	CON	CN		
Capric acid (C10:0)	0.01	0.04	0.006	0.005
Undecanoic acid (C11:0)	0.13	0.11	0.009	0.27
Tridecanoic acid (C13:0)	0.11	0.09	0.009	0.457
Myristic acid (C14:0)	2.78	1.77	0.206	0.008
Tetradecenoic acid (C14:1)	0.27	0.23	0.016	0.156
Pentadecanoic acid (C15:0)	0.48	0.21	0.054	0.006
Pentadecenoic acid (C15:1)	0.81	1.80	0.189	0.003
Palmitic acid (C16:0)	24.61	23.24	0.406	0.092
Palmitoleic acid (C16:1)	2.05	1.71	0.103	0.107
Heptadecanoic acid (C17:0)	1.24	0.89	0.073	0.008
Heptadecenoic acid (C17:1)	1.02	0.93	0.049	0.342
Stearic acid (C18:0)	11.13	12.17	0.429	0.242
Elaidic acid (C18:1n9t)	4.16	3.13	0.216	0.01
Oleic acid (C18:1n9c)	37.97	38.90	0.503	0.38
Linolelaidic acid (C18:2n6t)	0.98	1.16	0.038	0.012
Linoleic acid (C18:2n6c)	4.89	5.51	0.139	0.018
Arachidic acid (C20:0)	0.11	0.08	0.015	0.356
γ-Linolenic acid (C18:3n6)	0.10	0.10	0.006	0.502
Eicosenoic acid (C20:1)	0.13	0.11	0.011	0.535
α-Linolenic acid (C18:3n3)	1.97	2.13	0.099	0.443
Heneicosanoic acid (C21:0)	0.37	0.54	0.042	0.035
Eicosadienoic acid (C20:2)	0.06	0.09	0.014	0.294
Behenic acid (C22:0)	0.24	0.33	0.027	0.108
Eicosatrienoic acid (C20:3n6)	0.09	0.09	0.005	0.991
Methyl sinapate (C22:1n9)	0.23	0.23	0.017	0.969
Eicosatrienoic acid (C20:3n3)	0.09	0.09	0.005	0.911
Arachidonic acid (C20:4n6)	1.82	2.06	0.081	0.144
Eicosapentaenoic acid (C20:5n3)	1.04	1.10	0.017	0.068
Docosahexaenoic acid (C22:6n3)	1.10	1.12	0.021	0.734
Saturated fatty acid (SFA)	41.21	39.48	0.629	0.181
Unsaturated fatty acid (UFA)	58.77	60.48	0.632	0.187
UFA/SFA	1.44	1.54	0.04	0.216
Monounsaturated fatty acid (MUFA)	46.64	47.04	0.454	0.68
Polyunsaturated fatty acid (PUFA)	12.13	13.45	0.32	0.034
n-3 PUFA	4.19	4.44	0.091	0.197
n-6 PUFA	7.88	8.92	0.238	0.021
n-3/n-6 PUFA	1.88	2.01	0.034	0.044

^a^ Treatment; CON means control group, CN means 3% calcium nitrate group. ^b^ SEM means standard error.

## Data Availability

The data will be made available from the corresponding author upon reasonable request.

## References

[1] Wu Z.L., Yang X., Zhang J., Wang W., Liu D., Hou B., Bai T., Zhang R., Zhang Y., Liu H. (2023). Effects of forage type on the rumen microbiota, growth performance, carcass traits, and meat quality in fattening goats. Front. Vet. Sci..

[2] Lee C., Araujo R.C., Koenig K.M., Beauchemin K.A. (2017). Effects of encapsulated nitrate on growth performance, carcass characteristics, nitrate residues in tissues, and enteric methane emissions in beef steers: Finishing phase. J. Anim. Sci..

[3] McAllister T.A., Cheng K.-J., Okine E.K., Mathison G.W. (1996). Dietary, environmental and microbiological aspects of methane production in ruminants. Can. J. Anim. Sci..

[4] Kang M.G., Kwak M.J., Kang A., Park J., Lee D.J., Mun J., Kim S., Mun D., Lee W., Choi H. (2025). Metagenome-based microbial metabolic strategies to mitigate ruminal methane emissions using Komagataeibacter-based symbiotics. Sci. Total Environ..

[5] Yanza Y.R., Szumacher-Strabel M., Lechniak D., Ślusarczyk S., Kolodziejski P., Patra A.K., Váradyová Z., Lisiak D., Vazirigohar M., Cieslak A. (2022). Dietary Coleus amboinicus Lour. decreases ruminal methanogenesis and biohydrogenation, and improves meat quality and fatty acid composition in longissimus thoracis muscle of lambs. J. Anim. Sci. Biotechnol..

[6] Sheppard A.M., van de Ligt J.L.G., Pillai P., Crincoli C.M., Faris R.J., McGhee M.L., Frederick B.R. (2024). Safety of dietary nitrate supplementation by calcium nitrate for finishing pigs as measured by methemoglobin and serum and tissue nitrate levels. Transl. Anim. Sci..

[7] Schrenk D., Bignami M., Bodin L., Chipman J.K., Del Mazo J., Grasl-Kraupp B., Hoogenboom L.R., Leblanc J.C., Nebbia C.S., Nielsen E. (2020). Risk assessment of nitrate and nitrite in feed. EFSA J..

[8] Popova M., Guyader J., Silberberg M., Seradj A.R., Saro C., Bernard A., Gérard C., Martin C., Morgavi D.P. (2019). Changes in the Rumen Microbiota of Cows in Response to Dietary Supplementation with Nitrate, Linseed, and Saponin Alone or in Combination. Appl. Environ. Microbiol..

[9] den Besten G., van Eunen K., Groen A.K., Venema K., Reijngoud D.J., Bakker B.M. (2013). The role of short-chain fatty acids in the interplay between diet, gut microbiota, and host energy metabolism. J. Lipid Res..

[10] Yang Z., Alvarez B.V., Chakarova C., Jiang L., Karan G., Frederick J.M., Zhao Y., Sauvé Y., Li X., Zrenner E. (2005). Mutant carbonic anhydrase 4 impairs pH regulation and causes retinal photoreceptor degeneration. Hum. Mol. Genet..

[11] O’Fallon J.V., Busboom J.R., Nelson M.L., Gaskins C.T. (2007). A direct method for fatty acid methyl ester synthesis: Application to wet meat tissues, oils, and feedstuffs. J. Anim. Sci..

[12] Yang X., Liu T., Zhou J., An L., Pan F., Zhang H., Wang X., Xu G., Zheng C. (2025). Effects of Bacillus subtilis addition to milk replacer on growth performance, nutrient digestibility, intestinal microbiota, and short-chain fatty acid concentration of Hu lambs. Anim. Feed Sci. Technol..

[13] Tang L., Li Y., Srivathsan A., Gao Y., Li K., Hu D., Zhang D. (2020). Gut Microbiomes of Endangered Przewalski’s Horse Populations in Short- and Long-Term Captivity: Implication for Species Reintroduction Based on the Soft-Release Strategy. Front. Microbiol..

[14] Magoč T., Salzberg S.L. (2011). FLASH: Fast length adjustment of short reads to improve genome assemblies. Bioinformatics.

[15] Caporaso J.G., Kuczynski J., Stombaugh J., Bittinger K., Bushman F.D., Costello E.K., Fierer N., Peña A.G., Goodrich J.K., Gordon J.I. (2010). QIIME allows analysis of high-throughput community sequencing data. Nat. Methods.

[16] Bokulich N.A., Subramanian S., Faith J.J., Gevers D., Gordon J.I., Knight R., Mills D.A., Caporaso J.G. (2012). Quality-filtering vastly improves diversity estimates from Illumina amplicon sequencing. Nat. Methods.

[17] Rognes T., Flouri T., Nichols B., Quince C., Mahé F. (2016). VSEARCH: A versatile open source tool for metagenomics. PeerJ.

[18] Edgar R.C. (2013). UPARSE: Highly accurate OTU sequences from microbial amplicon reads. Nat. Methods.

[19] Quast C., Pruesse E., Yilmaz P., Gerken J., Schweer T., Yarza P., Peplies J., Glöckner F.O. (2013). The SILVA ribosomal RNA gene database project: Improved data processing and web-based tools. Nucleic Acids Res..

[20] Edgar R.C. (2004). MUSCLE: Multiple sequence alignment with high accuracy and high throughput. Nucleic Acids Res..

[21] Zhang J., Min L., Chang J., Ding S., Chi Y., Wang S., Ji S. (2025). Effects of perfluorolauric acid exposure on intestinal microbial community and physiological health indicators in mice. Sci. Rep..

[22] Yang X., Cao Q., Ma B., Xia Y., Liu M., Tian J., Chen J., Su C., Duan X. (2023). Probiotic powder ameliorates colorectal cancer by regulating Bifidobacterium animalis, Clostridium cocleatum, and immune cell composition. PLoS ONE.

[23] Araujo R.C., Pereira M.L.R., Couto V.R.M., Lemos B.J.M., Jorge da Cunha P.H., Arnhold E., Silva J.A., Fernandes J.J.R. (2021). Dose-response effect of encapsulated nitrate replacing soybean meal on growth performance, ingestive behavior, and blood metabolites of feedlot finishing bulls. Livest. Sci..

[24] Hughes J.M., Oiseth S.K., Purslow P.P., Warner R.D. (2014). A structural approach to understanding the interactions between colour, water-holding capacity and tenderness. Meat Sci..

[25] Hegarty R.S., Miller J., Oelbrandt N., Li L., Luijben J.P.M., Robinson D.L., Nolan J.V., Perdok H.B. (2017). Feed intake, growth, and body and carcass attributes of feedlot steers supplemented with two levels of calcium nitrate or urea. J. Anim. Sci..

[26] Scollan N.D., Enser M., Gulati S.K., Richardson I., Wood J.D. (2003). Effects of including a ruminally protected lipid supplement in the diet on the fatty acid composition of beef muscle. Br. J. Nutr..

[27] Cañeque V., Díaz M.T., Álvarez I., Lauzurica S., Pérez C., De la Fuente J. (2005). The influences of carcass weight and depot on the fatty acid composition of fats of suckling Manchego lambs. Meat Sci..

[28] Nuernberg K., Fischer A., Nuernberg G., Ender K., Dannenberger D. (2008). Meat quality and fatty acid composition of lipids in muscle and fatty tissue of Skudde lambs fed grass versus concentrate. Small Rumin. Res..

[29] Abbadi A., Domergue F., Bauer J., Napier J.A., Welti R., Zähringer U., Cirpus P., Heinz E. (2004). Biosynthesis of very-long-chain polyunsaturated fatty acids in transgenic oilseeds: Constraints on their accumulation. Plant Cell.

[30] Pascual J.V., Rafecas M., Canela M.A., Boatella J., Bou R., Baucells M.D., Codony R. (2007). Effect of increasing amounts of a linoleic-rich dietary fat on the fat composition of four pig breeds. Part III: Triacylglycerol composition in muscle and fat tissues. Food Chem..

[31] Scollan N.D., Dannenberger D., Nuernberg K., Richardson I., MacKintosh S., Hocquette J.-F., Moloney A.P. (2014). Enhancing the nutritional and health value of beef lipids and their relationship with meat quality. Meat Sci..

[32] Jump B.D. (2002). Dietary polyunsaturated fatty acids and regulation of gene transcription. Curr. Opin. Lipidol..

[33] Alizadeh A., Shahneh A.Z., Yousefi A.R., Omran M.H., Campbell A.W. (2013). Determining the effect of the fat-tail and carcass weight on meat fatty acid composition of Iranian lambs. Small Rumin. Res..

[34] Enser M., Hallett K., Hewitt B., Fursey G.A.J., Wood J.D. (1996). Fatty acid content and composition of english beef, lamb and pork at retail. Meat Sci..

[35] Lee H.H., Kim H., Park Y.L., Horn M.A., Kim J., Lee J., Toyoda S., Yun J., Kang H., Kim S.Y. (2025). Exploring Sulfate as an Alternative Electron Acceptor: A Potential Strategy to Mitigate N_2_O Emissions in Upland Arable Soils. Glob. Change Biol..

[36] Lee C., Araujo R.C., Koenig K.M., Beauchemin K.A. (2017). Effects of encapsulated nitrate on growth performance, nitrate toxicity, and enteric methane emissions in beef steers: Backgrounding phase. J. Anim. Sci..

[37] Liu J., Bai Y., Liu F., Kohn R.A., Tadesse D.A., Sarria S., Li R.W., Song J. (2022). Rumen Microbial Predictors for Short-Chain Fatty Acid Levels and the Grass-Fed Regimen in Angus Cattle. Animals.

[38] Zhao L., Meng Q., Ren L., Liu W., Zhang X., Huo Y., Zhou Z. (2015). Effects of Nitrate Addition on Rumen Fermentation, Bacterial Biodiversity and Abundance. Anim. Biosci..

[39] Ma G., Jin W., Zhang Y., Gai Y., Tang W., Guo L., Azzaz H.H., Ghaffari M.H., Gu Z., Mao S. (2024). A Meta-Analysis of Dietary Inhibitors for Reducing Methane Emissions via Modulating Rumen Microbiota in Ruminants. J. Nutr..

[40] Perumbakkam S., Craig A.M. (2012). Biochemical and Microbial Analysis of Ovine Rumen Fluid Incubated with 1,3,5-Trinitro-1,3,5-triazacyclohexane (RDX). Curr. Microbiol..

[41] Marais J.P., Therion J.J., Mackie R.I., Kistner A., Dennison C. (2005). Effect of nitrate and its reduction products on the growth and activity of the rumen microbial population. Br. J. Nutr..

[42] Asanuma N., Yokoyama S., Hino T. (2014). Effects of nitrate addition to a diet on fermentation and microbial populations in the rumen of goats, with special reference to Selenomonas ruminantium having the ability to reduce nitrate and nitrite. Anim. Sci. J..

[43] Bharanidharan R., Tomple B.M., Lee J., Nirmal Athauda A.A.K., Huh S., Hong W., Kim N.Y., Lim D.H., Kim J.G., Kim K.H. (2025). Effects of Dietary Supplementation of Nitrate on Enteric Methane Production, Performance and Rumen Microbiome of Hanwoo Steers. J. Anim. Sci..

[44] Patra A.K., Yu Z. (2014). Combinations of nitrate, saponin, and sulfate additively reduce methane production by rumen cultures in vitro while not adversely affecting feed digestion, fermentation or microbial communities. Bioresour. Technol..

[45] Zhou Z., Yu Z., Meng Q. (2011). Effects of nitrate on methane production, fermentation, and microbial populations in in vitro ruminal cultures. Bioresour. Technol..

[46] Granja-Salcedo Y.T., Fernandes R.M., Araujo R.C.d., Kishi L.T., Berchielli T.T., Resende F.D.d., Berndt A., Siqueira G.R. (2019). Long-Term Encapsulated Nitrate Supplementation Modulates Rumen Microbial Diversity and Rumen Fermentation to Reduce Methane Emission in Grazing Steers. Front. Microbiol..

[47] Salleron L., Magistrelli G., Mary C., Fischer N., Bairoch A., Lane L. (2014). DERA is the human deoxyribose phosphate aldolase and is involved in stress response. Biochim. Biophys. Acta.

[48] Mei Z., Shen Z., Pu J., Liu Q., Liu G., He X., Wang Y., Yue J., Ge S., Li T. (2024). NAT10 mediated ac4C acetylation driven m6A modification via involvement of YTHDC1-LDHA/PFKM regulates glycolysis and promotes osteosarcoma. Cell Commun. Signal..

[49] Similä M.E., Auranen M., Piirilä P.L. (2020). Beneficial Effects of Ketogenic Diet on Phosphofructokinase Deficiency (Glycogen Storage Disease Type VII). Front. Neurol..

[50] Jin Y., Penning T.M. (2006). Aldo-keto reductases and bioactivation/detoxication. Annu. Rev. Pharmacol. Toxicol..

[51] Lu S.C. (2013). Glutathione synthesis. Biochim. Biophys. Acta (BBA)-Gen. Subj..

[52] Lindstad R.I., Hermansen L.F., McKinley-McKee J.S. (1992). The kinetic mechanism of sheep liver sorbitol dehydrogenase. Eur. J. Biochem..

[53] Huang C.-L., Moe O.W. (2011). Klotho: A novel regulator of calcium and phosphorus homeostasis. Pflügers Arch.-Eur. J. Physiol..

[54] Sala D., Marchet S., Nanetti L., Legati A., Mariotti C., Lamantea E., Ghezzi D., Catania A., Lamperti C. (2024). A novel MT-ATP6 variant associated with complicated ataxia in two unrelated Italian patients: Case report and functional studies. Orphanet J. Rare Dis..

[55] Ferraris R.P. (2001). Dietary and developmental regulation of intestinal sugar transport. Biochem. J..

[56] Wolfe A.J. (2005). The acetate switch. Microbiol. Mol. Biol. Rev..

